# A multicenter prospective cohort study developing and validating a SIRI-based machine learning model and simplified risk score for predicting postherpetic neuralgia

**DOI:** 10.3389/fimmu.2026.1868650

**Published:** 2026-06-05

**Authors:** Mengying Mao, Fangzheng Cao, Yongxing Yan, Huili Liu, Wenjing Wu, Bin Xu

**Affiliations:** 1 The Second School of Clinical Medicine, Zhejiang Chinese Medical University, Hangzhou, China; 2 Department of Neurology, The Second Affiliated Hospital of Zhejiang Chinese Medical University (Zhejiang Xinhua Hospital), Hangzhou, China; 3 Department of Neurology, Hangzhou Third People’s Hospital, Hangzhou, China

**Keywords:** extreme gradient boosting, machine learning, postherpetic neuralgia, prediction model, prospective cohort study, risk scoring, systemic inflammatory response index

## Abstract

**Background:**

Postherpetic neuralgia (PHN) is the most common and severe complication of herpes zoster (HZ), and early identification of high-risk patients remains a major clinical challenge. The systemic inflammatory response index (SIRI) is a novel inflammatory marker that has demonstrated prognostic value in multiple diseases, but its association with the risk of PHN development has not been reported to date. Existing PHN prediction models mostly rely on traditional regression methods with limited predictive performance, and lack clinically practical risk stratification tools.

**Methods:**

After screening for eligibility and follow-up completion, 1135 patients from the Hangzhou Third People’s Hospital and 226 patients from the Second Affiliated Hospital of Zhejiang Chinese Medical University were included in the final statistical analysis. 1135 enrolled patients were stratified and randomly split at a 3:1 ratio into a training set for model development and an internal test set for internal validation, while data from 226 enrolled patients served as the independent external validation set. A total of 36 candidate predictive factors were first screened via univariate Logistic regression (LR) and collinearity diagnosis, followed by Least Absolute Shrinkage and Selection Operator (LASSO) regression with 10-fold cross-validation to identify core predictive factors. Finally, the predictive performance of 8 machine learning algorithms was compared, and a simplified risk scoring table was constructed based on SHapley Additive exPlanations (SHAP) analysis.

**Results:**

A total of 1361 patients were finally included, with 511 (37.55%) developing PHN during follow-up. Multivariate binary LR confirmed that SIRI was an independent predictive factor for PHN (odds ratio [OR] = 1.448, 95% confidence interval [CI] 1.119–1.874, *P* = 0.005). LASSO regression identified 6 core predictive variables: age, SIRI, numerical rating scale (NRS) score, time to treatment, rash location, and neutrophil-to-albumin ratio (NAR). The eXtreme Gradient Boosting (XGBoost) model exhibited the optimal predictive performance, with area under the receiver operating characteristic curve (AUC) values of 0.889 (95% CI 0.868–0.910) in the training set, 0.857 (95% CI 0.813–0.901) in the internal test set, and 0.900 (95% CI 0.860–0.940) in the external validation set. SHAP analysis revealed that SIRI ranked second in feature contribution to the model (20.1%), and the simplified risk scoring table constructed based on SHAP values achieved an AUC of 0.904 (95% CI 0.867–0.941) in the external validation set.

**Conclusion:**

This study is the first to confirm that SIRI is an independent predictive biomarker for PHN development. The SIRI-based XGBoost model demonstrates excellent predictive performance, and the developed simplified risk scoring table has high clinical practicability, in which, early active intervention is recommended for patients with a risk score ≥18 to reduce the incidence of PHN.

## Introduction

Herpes zoster (HZ) is an acute infectious disease caused by the reactivation of varicella-zoster virus (VZV) (1). Its most common and devastating complication is postherpetic neuralgia (PHN), which is internationally defined as neuropathic pain persisting for more than 3 months after the onset of HZ rash (2). The pain caused by PHN is mostly burning, electric shock-like, or stabbing, which can last for months to years, seriously impairing daily activity ability of patients. Epidemiological data show that the annual incidence of HZ in China is approximately 7.7% (3), and about 29.8% of HZ patients progress to PHN (4), indicating a large population of PHN patients and an urgent clinical need for prevention and treatment. Therefore, early identification of high-risk HZ patients who may develop PHN is crucial for improving patient prognosis and reducing the risk of PHN. However, there remains a lack of efficient and convenient tools in clinical practice to accurately identify high-risk PHN patients. Traditional prediction methods mainly rely on single clinical features such as advanced age, severe acute pain, and large-area skin lesions (5, 6). Although these indicators have certain predictive value, their sensitivity and specificity are limited. The systemic inflammatory response index (SIRI), calculated as (neutrophil count × monocyte count)/lymphocyte count, is a novel and easily calculable composite inflammatory indicator. SIRI simultaneously integrates two innate immune effector cells (neutrophils and monocytes) and the adaptive immune component (lymphocytes), which exactly cover the core pathophysiological links in the development and progression of PHN. Compared with traditional indicators such as Neutrophil-to-Lymphocyte Ratio (NLR) and C-reactive protein-to-lymphocyte ratio (CLR), SIRI can more comprehensively assess the body’s inflammatory and immune homeostasis. Currently, SIRI has demonstrated good application value in the prognostic prediction of cardiovascular diseases, stroke-associated pneumonia, and various malignant tumors (7–9). However, to date, the association between SIRI and PHN development risk has not been reported in the literature, representing a research gap. Machine learning algorithms, such as Random Forest (RF) and eXtreme Gradient Boosting (XGBoost), have been increasingly and widely applied in the field of medical prognostic prediction (10–12). Compared with traditional statistical models, they can automatically capture complex relationships in data, usually achieving higher prediction accuracy. With the help of interpretability techniques such as SHapley Additive exPlanations (SHAP) (13), the clinical translation barrier of machine learning models can be effectively addressed, enabling clinicians to clearly understand the contribution of each predictive factor to the PHN risk of individual patients. However, most existing PHN prediction models are developed based on traditional Logistic regression (LR) models with single-center retrospective cohorts (5, 6), lacking independent external validation, with predictive area under the receiver operating characteristic curve (AUC) values mostly ranging from 0.70 to 0.80 and limited predictive performance (5, 6, 14, 15).

Our study innovatively introduces SIRI as a predictive factor, systematically evaluates the independent predictive value of SIRI for PHN development based on a multicenter prospective cohort design, integrates SIRI and other laboratory indicators with routine clinical features, constructs and internally and externally validates a PHN risk prediction model using machine learning algorithms, and ultimately developed a visualized, easily promotable simplified risk scoring tool based on interpretability analysis.

## Materials and methods

### Study design

**Figure 1:**
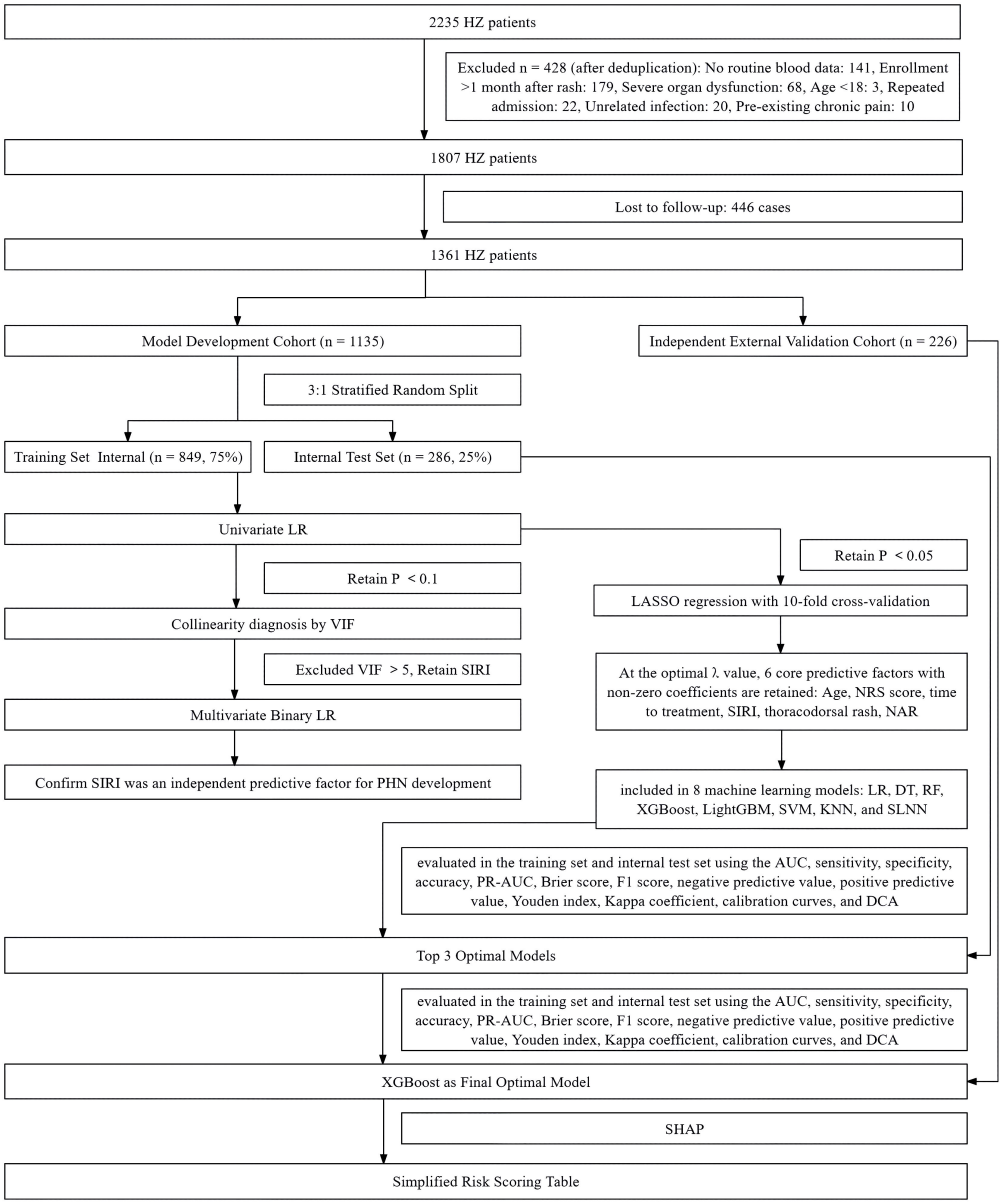
Flow diagram of the study design. HZ, herpes zoster; ZCMU, Zhejiang Chinese Medical University; PHN, postherpetic neuralgia; LR, logistic regression; VIF, variance inflation factor; LASSO, least absolute shrinkage and selection operator; DT, decision tree; RF, random forest; XGBoost: extreme gradient boosting; LightGBM, light gradient boosting machine; SVM, support vector machine; KNN, k-nearest neighbor; SLNN, single hidden-layer neural network; AUC, area under the receiver operating characteristic curve; DCA, decision curve analysis.

This study was reported in strict adherence to the Transparent Reporting of a multivariable prediction model for Individual Prognosis Or Diagnosis (TRIPOD) statement. This was a multicenter, prospective, observational cohort study conducted in strict accordance with the ethical principles of the Declaration of Helsinki. The study protocol was approved by the Ethics Committees of the leading institution, the Second Affiliated Hospital of Zhejiang Chinese Medical University (approval number: 2025-Yan-169-IH01), as well as the Ethics Committees of the participating institutions, Hangzhou Third People’s Hospital (approval number: 2023KA033). A total of 2235 acute HZ patients were initially recruited from the two centers: 1933 patients from the first center (January 1 to June 30, 2025) and 302 patients from the second center (October 1 to December 31, 2025). After eligibility screening and follow-up, 1135 and 226 patients from each center respectively were included in the final analysis. The 1135 patients constituted the model development cohort for model training and internal validation, the other 226 patients served as the independent external validation cohort to evaluate the generalizability of the model. The prognostic prediction time window of this study was from patient baseline enrollment (within 1 month after the onset of HZ rash) to 90 ± 7 days after rash onset, with the primary outcome being the development of PHN during this time window. The detailed study design presented in **Figure 1**.

### Inclusion and exclusion criteria

Inclusion criteria: (1) Age ≥18 years; (2) Meeting the clinical diagnostic criteria for acute HZ; (3) Enrollment within 1 month after rash onset; (4) Having relatively complete baseline clinical data and laboratory test results; (5) Agreeing to and being able to cooperate with 3 months of standardized follow-up.

Exclusion criteria: (1) Presence of severe heart, liver, or kidney dysfunction (definition: severe heart dysfunction refers to left ventricular ejection fraction <40% or New York Heart Association (NYHA) class III–IV; severe liver dysfunction refers to alanine transaminase or aspartate transaminase >3 times the upper limit of normal or Model for End-Stage Liver Disease (MELD) score ≥15; severe kidney dysfunction refers to estimated glomerular filtration rate (eGFR) <30 ml·min^-^¹·1.73m^-^²); (2) Pre-existing chronic painful diseases before HZ onset (such as cancer pain, diabetic peripheral neuropathy, etc.); (3) Acute infectious diseases unrelated to HZ within 1 month before enrollment, or received systemic antiviral, analgesic, glucocorticoid, or other targeted treatments for HZ before the first visit; (4) Pregnant or lactating women; (5) Presence of language barriers, mental illnesses, or other factors preventing cooperation with telephone follow-up; (6) Refusal to sign written informed consent.

### Baseline data collection

Baseline information was collected from the electronic medical record systems of each center by 2 uniformly trained researchers using standardized data forms. Routine blood tests at the two centers were performed using standardized testing procedures with calibrated automatic hematology analyzers. All baseline data were collected within 24 hours of the patient’s first visit and before receiving any systemic treatments (including antiviral drugs, analgesics, glucocorticoids, and other targeted therapies for HZ). All serological parameters were the results of the first laboratory test using venous blood collected from the antecubital vein after the patient fasted for 8 hours. Composite inflammatory indicators were calculated based on routine blood and biochemical test results as follows: SIRI = neutrophil count × monocyte count/lymphocyte count; NLR = neutrophil count/lymphocyte count; CLR = high-sensitivity C-reactive protein (hs-CRP)/lymphocyte count; neutrophil-to-albumin ratio (NAR) = neutrophil percentage/albumin concentration. The detailed collected content is shown in **Table 1**.

**Table 1:** Comparison of baseline data between PHN group and non-PHN group.

Characteristic		Non-PHN group (*n* = 850)	PHN group (*n* = 511)	P value
Age (years)		59.99 ± 14.04	67.75 ± 10.26	<0.001
NAR		1.71 ± 0.35	1.82 ± 0.40	0.018
Complement C3 (g/L)		1.14 ± 0.20	1.13 ± 0.20	0.319
Complement C4 (g/L)		0.38 ± 0.12	0.39 ± 0.12	0.346
Globulin (g/L)		26.44 ± 3.51	26.07 ± 3.79	0.031
LDL (mmol/L)		2.81 ± 0.90	2.70 ± 0.88	0.758
Neutrophil percentage (%)		62.81 ± 12.22	64.04 ± 12.02	0.097
Albumin (g/L)		36.99 ± 3.20	35.6 ± 3.54	<0.001
Time to treatment (days)		5 (4, 7)	7 (5, 20)	<0.001
hs-CRP (mg/L)		0.5 (0.5, 2.5)	0.7 (0.5, 4.8)	0.308
Lymphocyte count (×10^9^/L)		1.37 (1.01, 1.83)	1.37 (0.92, 1.75)	0.297
Neutrophil count (×10^9^/L)		3.31 (2.47, 4.45)	3.41 (2.60, 4.6)	0.083
SIRI		0.88 (0.58, 1.44)	1.09 (0.67, 1.83)	<0.001
NSE (ng/mL)		9.49 (8.29, 10.7)	9.46 (8.14, 11.01)	0.114
Creatinine (μmol/L)		61 (51, 72)	63 (54, 76.75)	0.001
HDL (mmol/L)		1.27 (1.05, 1.5)	1.23 (1.03, 1.46)	0.237
VZV-IgA (g/L)		2.3 (1.67, 2.97)	2.26 (1.65, 2.95)	0.508
VZV-IgG (g/L)		13.54 (11.67, 15.62)	13.12 (10.70, 15.82)	0.045
VZV-IgM (g/L)		0.82 (0.54, 1.22)	0.81 (0.51, 1.24)	0.849
WBC (×10^9^/L)		5.3 (4.4, 6.7)	5.5 (4.43, 6.9)	0.161
Eosinophil percentage (%)		1.3 (0.2, 2.6)	1.2 (0.3, 2.4)	0.775
Monocyte count (×10^9^/L)		0.41 (0.3, 0.54)	0.44 (0.33, 0.57)	<0.001
Eosinophil count (×10^9^/L)		0.06 (0.01, 0.13)	0.06 (0.02, 0.13)	0.765
NLR		2.38 (1.59, 3.74)	2.54 (1.71, 4.12)	0.034
CLR		0.57 (0.29, 2.05)	0.71 (0.26, 4.28)	0.192
Sex, n (%)	Male	375 (44.1)	242 (47.4)	0.056
	Female	475 (55.9)	269 (52.6)	
NRS score, n (%)	0	1 (0.1)	0 (0)	<0.001
	1	95 (11.2)	0 (0)	
	2	375 (44.1)	9 (1.8)	
	3	259 (30.5)	31 (6.1)	
	4	59 (6.9)	180 (35.2)	
	5	34 (4.0)	131 (25.6)	
	6	20 (2.4)	63 (12.3)	
	7	7 (0.8)	52 (10.2)	
	8	0 (0)	29 (5.7)	
	9	0 (0)	14 (2.7)	
	10	0 (0)	2 (0.4)	
Prodromal pain, n (%)	No	589 (69.3)	317 (62.0)	0.006
	Yes	261 (30.7)	194 (38.0)	
Rash location, n (%)	Cephalic-facial	285 (33.5)	128 (25.0)	<0.001
	Thoracodorsal	203 (23.9)	185 (36.2)	
	Lumbabdominal	164 (19.3)	102 (20.0)	
	Cervicoscapular	54 (6.4)	21 (4.1)	
	Upper limb	43 (5.1)	34 (6.7)	
	Lower limb	101 (11.9)	41 (8.0)	
Hypertension, n (%)	No	556 (65.4)	281 (55.0)	<0.001
	Yes	294 (34.6)	230 (45.0)	
Diabetes, n (%)	No	744 (87.5)	413 (80.8)	0.001
	Yes	106 (12.5)	98 (19.2)	
Malignancy, COPD or CKD, n (%)	No	733 (86.2)	444 (86.9)	0.544
	Yes	117 (13.8)	67 (13.1)	
Antidepressant or anxiolytic medication use, n (%)	No	826 (97.2)	484 (94.7)	0.031
	Yes	24 (2.8)	27 (5.3)	
Anxiety or depression, n (%)	No	835 (98.2)	499 (97.7)	0.233
	Yes	15 (1.8)	12 (2.3)	
Sleep disorders, n (%)	No	445 (52.4)	192 (37.6)	<0.001
	Yes	405 (47.6)	319 (62.4)	
Immunosuppressive status, n (%)	No	812 (95.5)	478 (93.5)	0.009
	Yes	38 (4.5)	33 (6.5)	

NAR, neutrophil-to-albumin ratio; LDL, low-density lipoprotein; HDL, high-density lipoprotein; hs-CRP, high-sensitivity C-reactive protein; SIRI, systemic inflammation response index; NSE, neuron-specific enolase; VZV, varicella-zoster virus; IgA, immunoglobulin A; IgG, immunoglobulin G; IgM, immunoglobulin M; WBC, white blood cell; NLR, neutrophil-to-lymphocyte ratio; CLR, C-reactive protein-to-lymphocyte ratio; NRS, numerical rating scale; COPD, chronic obstructive pulmonary disease; CKD, chronic kidney disease.

### Data processing

All statistical analyses were performed using SPSS 26 and R 4.3.0 software. SPSS was used for missing value imputation, baseline data comparison, univariate and multivariate LR analysis, while R software was used for Least Absolute Shrinkage and Selection Operator (LASSO) regression, machine learning model construction, interpretability analysis, and visualization. The significance level α was set at 0.05 for two-tailed tests. The 36 prespecified candidate variables were uniformly standardized and coded: binary variables were assigned yes = 1 and no = 0; sex was coded as female = 1 and male = 0; rash location, as an unordered categorical variable, was coded using dummy variables with the cephalic-facial region as the reference group; the outcome variable was coded as PHN developed = 1 and PHN not developed = 0. In this study, three continuous variables (NSE, hs-CRP and albumin) had missing data with missing rates of 13.22%, 2.03%, and 3.79%, respectively. All variables had missing rates <20%, and multiple imputation by chained equations (MICE) with 5 imputations and 10 iterations was used for data completion. Variables with missing rates >20% were directly excluded from the candidate variables, and there were no such variables in this study.

### Statistical analysis, model development and construction of risk scoring table

First, the baseline characteristics of 1361 patients were compared. Normality of continuous data was tested using the Shapiro-Wilk test, histograms, and Q-Q plots; P >0.05 was considered normally distributed. Normally distributed measurement data were expressed as mean ± standard deviation, and intergroup comparisons were performed using independent samples t-test; non-normally distributed measurement data were expressed as median (P25, P75), and intergroup comparisons were performed using the Mann-Whitney U test; categorical data were expressed as counts (percentages), and intergroup comparisons were performed using the chi-square test or Fisher’s exact test. Second, data from 1135 patients in the model development cohort were stratified and randomly split at a 3:1 ratio into a training set (n = 849, 75%) and an internal test set (n = 286, 25%). To strictly avoid information leakage and ensure the reliability of model performance evaluation, all data-dependent preprocessing and model development steps (including univariate screening, collinearity diagnosis, LASSO variable selection, hyperparameter tuning, and SHAP interpretability analysis) were performed exclusively in the training set. The internal test set and independent external validation set were only used for final model performance evaluation and were not involved in any model development process. Potential influencing factors with P <0.1 were screened via univariate LR in the training set. Collinearity diagnosis was performed on the screened variables by calculating the variance inflation factor (VIF), and variables with VIF >5 were excluded as highly collinear. The above-screened indicators were included in multivariate binary LR analysis to confirm whether SIRI was an independent predictive factor for PHN development. Furthermore, all variables with P <0.05 in the univariate analysis within the training set were included in LASSO regression with 10-fold cross-validation. The optimal penalty factor λ was selected based on the 1-standard error (1-se) rule to screen core predictive variables with non-zero coefficients. The screened core predictive variables were respectively included in 8 machine learning models: LR, decision tree (DT), RF, XGBoost, Light Gradient Boosting Machine (LightGBM), support vector machine (SVM), K-nearest neighbor algorithm (KNN), and single hidden layer neural network (SLNN). All models were optimized via hyperparameter grid search with 10-fold cross-validation, and a random seed of 1234 was set to ensure reproducibility. The hyperparameter search ranges and adapted packages for 8 models are shown in **Supplementary Table 1**. The preliminary performance of the 8 models was evaluated in the training set and internal test set. The discriminative ability of the models was evaluated using the AUC, sensitivity, specificity, accuracy, PR-AUC, Brier score, F1 score, negative predictive value, positive predictive value, Youden index, and Kappa coefficient. The calibration and clinical net benefit of the models were evaluated using calibration curves and decision curve analysis (DCA). Based on the preliminary evaluation results, the top 3 models with the best performance were selected for final generalizability validation using the independent external validation set. Finally, based on the optimal model, the incremental predictive value of SIRI was evaluated, followed by the use of SHAP to analyze the contribution of each variable, and a visualized simplified clinical risk scoring table was constructed based on SHAP values and optimal cutoff values.

## Results

### Baseline characteristics of the study population

A total of 1361 patients with acute HZ from two centers were finally included in this study, including 617 males (45.33%) and 744 females (54.67%), with a mean age of (62.90 ± 12.62) years. At the end of follow-up, 511 (37.55%) patients progressed to PHN, and 850 (62.45%) patients did not develop PHN.

Intergroup comparison of baseline data showed statistically significant differences between the PHN group and non-PHN group in terms of age, NAR, globulin, albumin, time to treatment, SIRI, creatinine, VZV-IgG, monocyte count, NLR, NRS score, prodromal pain, rash location, hypertension, diabetes, antidepressant or anxiolytic medication use, sleep disorders, and immunosuppressive status (all *P* < 0.05), as shown in **Table 1**. After Bonferroni correction, statistically significant differences were still observed in age, albumin, time to treatment, SIRI, creatinine, monocyte count, NRS score, rash location, hypertension, diabetes, sleep disorders, indicating robust between-group differences in these variables (α = 0.0014). During the 3-month follow-up period, 11.46% (156/1361) of patients received special pain intervention treatments, including stellate ganglion block, spinal nerve root radiofrequency ablation, facial nerve decompression, pulsed radiofrequency, stimulator lead implantation or replacement, and trigeminal nerve radiofrequency ablation. The incidence of special interventions was significantly higher in the PHN group (19.0%, 97/511) than in the non-PHN group (6.9%, 59/850) (*P* < 0.001), which is consistent with clinical practice: patients with more severe acute pain and higher baseline PHN risk are more likely to receive aggressive interventional pain management.

### Screening of predictive factors and independent predictive value of SIRI

The comparison of baseline data between the training set and the test set showed that there was no statistically significant difference in all characteristics between the two groups (*P >*0.05), as shown in **Table 2**. The distribution of special pain interventions during follow-up was also balanced between the training set (10.1%, 85/849) and the internal test set (11.2%, 32/286) (*P* = 0.715), indicating no systematic bias between the two subsets used for model development and internal validation.

**Table 2:** Comparison of baseline characteristics between the training set and the test set.

Characteristic		Training set (*n* = 849)	Test set (*n* = 286)	P value
Age (years)		63.08 ± 13.34	62.74 ± 13.02	0.708
NAR		1.75 ± 0.37	1.75 ± 0.38	0.884
Complement C3 (g/L)		1.13 ± 0.20	1.15 ± 0.22	0.166
Complement C4 (g/L)		0.38 ± 0.12	0.39 ± 0.12	0.698
Globulin (g/L)		26.33 ± 3.65	26.18 ± 3.54	0.530
HDL (mmol/L)		1.28 ± 0.35	1.30 ± 0.35	0.538
VZV-IgA (g/L)		2.37 ± 0.97	2.45 ± 1.10	0.232
VZV-IgG (g/L)		13.86 ± 3.69	13.47 ± 3.40	0.109
LDL (mmol/L)		2.76 ± 0.89	2.77 ± 0.91	0.964
Neutrophil percentage (%)		63.00 ± 12.18	64.16 ± 12.04	0.959
Albumin (g/L)		36.46 ± 3.48	36.44 ± 3.17	0.091
Eosinophil percentage (%)		1.77 ± 1.98	1.59 ± 2.03	0.682
Time to treatment (days)		6 (4, 10)	6 (4, 10)	0.997
Eosinophil count (×10^9^/L)		0.07 (0.01, 0.13)	0.06 (0.01, 0.13)	0.441
hs-CRP (mg/L)		0.5 (0.5, 3.7)	0.7(0.5,3.2)	0.540
WBC (×10^9^/L)		5.4 (4.4, 6.7)	5.4(4.4,6.75)	0.996
Lymphocyte count (×10^9^/L)		1.38 (1, 1.82)	1.31(0.91,1.755)	0.140
Monocyte count (×10^9^/L)		0.42 (0.31, 0.55)	0.41(0.3,0.535)	0.305
Neutrophil count (×10^9^/L)		3.31(2.53, 4.48)	3.46(2.54,4.67)	0.476
NLR		2.4(1.61,3.8925)	2.6(1.63,4.07)	0.138
CLR		0.58(0.28,2.695)	0.67(0.3,2.835)	0.410
SIRI		0.95 (0.6, 1.52)	1.08 (0.65, 1.72)	0.098
NSE (ng/mL)		9.55 (8.16, 10.87)	9.35 (8.35, 10.75)	0.554
Creatinine (μmol/L)		62 (53, 74)	60 (51, 72.5)	0.136
VZV-IgM (g/L)		0.82 (0.53, 1.21)	0.81 (0.54, 1.285)	0.450
Sex, n (%)	Male	385 (74.8)	130 (25.2)	0.925
	Female	465 (75)	155 (25)	
NRS score, n (%)	0	1 (100)	0 (0)	0.448
	1	52 (66.7)	26 (33.3)	
	2	243 (77.4)	71 (22.6)	
	3	173 (72.4)	66 (27.6)	
	4	155 (76.4)	48 (23.6)	
	5	112 (79.4)	29 (20.6)	
	6	49 (70)	21 (30)	
	7	39 (76.5)	12 (23.5)	
	8	18 (72)	7 (28)	
	9	7 (58.3)	5 (41.7)	
	10	1 (100)	0 (0)	
Prodromal pain, n (%)	No	570 (75.5)	185 (24.5)	0.506
	Yes	280 (73.7)	100 (26.3)	
Rash location, n (%)	Cephalic-facial	258 (75.2)	85 (24.8)	0.420
	Thoracodorsal	251 (77.2)	74 (22.8)	
	Lumbabdominal	163 (73.4)	59 (26.6)	
	Cervicoscapular	41 (66.1)	21 (33.9)	
	Upper limb	51 (79.7)	13 (20.3)	
	Lower limb	86 (72.3)	33 (27.7)	
Hypertension, n (%)	No	522 (74.9)	175 (25.1)	0.998
	Yes	328 (74.9)	110 (25.1)	
Diabetes, n (%)	No	724 (75.1)	240 (24.9)	0.693
	Yes	126 (73.7)	45 (26.3)	
Malignancy, COPD or CKD, n (%)	No	730 (74.4)	251 (25.6)	0.351
	Yes	120 (77.9)	34 (22.1)	
Antidepressant or anxiolytic medication use, n (%)	No	816 (74.7)	276 (25.3)	0.519
	Yes	34 (79.1)	9 (20.9)	
Anxiety or depression, n (%)	No	832 (74.8)	281 (25.2)	0.449
	Yes	18 (81.8)	4 (18.2)	
Sleep disorder, n (%)	No	394 (74.5)	135 (25.5)	0.766
	Yes	456 (75.2)	150 (24.8)	
Immunosuppressive status, n (%)	No	807 (75)	269 (25)	0.715
	Yes	43 (72.9)	16 (27.1)	

Univariate LR analysis showed that age, NRS score, time to treatment, rash location, hypertension, diabetes, sleep disorders, hs-CRP, neutrophil percentage, eosinophil percentage, lymphocyte count, monocyte count, neutrophil count, NLR, CLR, NAR, SIRI, albumin, creatinine, globulin, VZV-IgG, and LDL were potential influencing factors for PHN development (P < 0.05), as shown in **Table 3**.

**Table 3:** Univariate LR analysis result of PHN-related influencing factors.

Predictive variable	Regression coefficient B	OR value (95% CI)	P value
Sex	-0.184	0.832 (0.631, 1.098)	0.193
Age	0.047	1.048 (1.035, 1.061)	<0.001
NRS score	1.466	4.330 (3.568, 5.255)	<0.001
Prodromal pain	0.278	1.321 (0.987, 1.768)	0.061
Time to treatment	0.087	1.091 (1.069, 1.114)	<0.001
Rash location			<0.001
Thoracodorsal	0.704	2.022 (1.412, 2.897)	<0.001
Lumbabdominal	0.276	1.318 (0.875, 1.985)	0.186
Cervicoscapular	-0.403	0.668 (0.313, 1.427)	0.298
Upper limb	0.452	1.571 (0.852, 2.899)	0.148
Lower limb	-0.108	0.898 (0.529, 1.523)	0.689
Hypertension	0.352	1.421 (1.072, 1.885)	0.015
Diabetes	0.466	1.593 (1.089, 2.331)	0.016
Malignancy, COPD or CKD	-0.046	0.955 (0.641, 1.424)	0.821
Antidepressant or anxiolytic medication use	0.478	1.612 (0.811, 3.204)	0.173
Anxiety or depression	0.240	1.271 (0.496, 3.254)	0.617
Sleep disorder	0.600	1.823 (1.375, 2.416)	<0.001
Immunosuppressive status	0.532	1.703 (0.921, 3.148)	0.090
hs-CRP	0.032	1.032 (1.016, 1.050)	<0.001
WBC	0.059	1.061 (0.991, 1.137)	0.091
Neutrophil percentage	0.016	1.016 (1.004, 1.028)	0.007
Eosinophil percentage	-0.086	0.917 (0.851, 0.988)	0.024
Lymphocyte count	-0.235	0.791 (0.638, 0.979)	0.031
Monocyte count	1.369	3.930 (1.860, 8.305)	<0.001
Neutrophil count	0.114	1.121 (1.030, 1.219)	0.008
Eosinophil count	-1.054	0.349 (0.094, 1.286)	0.114
NLR	0.120	1.127 (1.058, 1.202)	<0.001
CLR	0.033	1.034 (1.016, 1.053)	<0.001
NAR	1.105	3.018 (2.034, 4.479)	<0.001
SIRI	0.318	1.374 (1.207, 1.564)	<0.001
NSE	0.058	1.059 (1.000, 1.122)	0.051
Albumin	-0.133	0.876 (0.838, 0.915)	<0.001
Complement C3	0.059	1.061 (0.557, 2.018)	0.858
Complement C4	0.778	2.177 (0.736, 6.440)	0.160
Creatinine	0.012	1.012 (1.004, 1.020)	0.004
Globulin	-0.039	0.962 (0.925, 0.999)	0.046
HDL	-0.142	0.867 (0.585, 1.284)	0.477
VZV-IgA	-0.083	0.921 (0.808, 1.049)	0.216
VZV-IgG	-0.044	0.957 (0.921, 0.994)	0.024
VZV-IgM	0.041	1.042 (0.848, 1.280)	0.696
LDL	-0.165	0.848 (0.724, 0.993)	0.041

OR, odds ratio; CI, confidence interval.

Collinearity diagnosis was performed on variables with *P* < 0.1 screened by univariate analysis, as shown in **Supplementary Table 2**. Variables with VIF >5 were excluded as highly collinear. The core study indicator SIRI was retained during the process. The results of the secondary collinearity diagnosis showed that all remaining variables had VIF <5, indicating no significant collinearity between the final included variables. Variables retained after collinearity diagnosis were included in multivariate binary LR analysis, and the results showed that SIRI was an independent predictive factor for PHN development (OR = 1.448, 95% CI 1.119–1.874, *P* = 0.005), as shown in **Table 4**.

**Table 4:** Multivariate binary LR analysis result of PHN-related influencing factors.

Predictive variable	Regression coefficient B	OR value (95% CI)	P value
Sex	-0.094	0.911 (0.531, 1.562)	0.734
Age	0.040	1.041 (1.021, 1.062)	<0.001
NRS score	1.683	5.380 (4.207, 6.879)	<0.001
Prodromal pain	-0.257	0.774 (0.486, 1.232)	0.280
Time to treatment	-0.025	0.975 (0.945, 1.007)	0.128
Rash location (reference: cephalic-facial)			0.366
Thoracodorsal	0.071	1.073 (0.479, 2.404)	0.863
Lumbabdominal	0.555	1.743 (0.809, 3.754)	0.156
Cervicoscapular	0.084	1.088 (0.475, 2.494)	0.842
Upper limb	-0.337	0.714 (0.211, 2.418)	0.588
Lower limb	0.064	1.066 (0.379, 2.998)	0.903
Hypertension	-0.482	0.618 (0.380, 1.003)	0.051
Diabetes	0.322	1.380 (0.718, 2.654)	0.334
Malignancy, COPD or CKD	-0.711	0.491 (0.253, 0.956)	0.036
Antidepressant or anxiolytic medication use	1.158	3.184 (0.905, 11.205)	0.071
Anxiety or depression	-0.481	0.618 (0.111, 3.439)	0.583
Sleep disorder	-0.749	0.473 (0.290, 0.770)	0.003
Immunosuppressive status	1.143	3.137 (1.108, 8.884)	0.031
SIRI	0.370	1.448 (1.119, 1.874)	0.005
NSE	-0.058	0.944 (0.860, 1.036)	0.223
Complement C3	0.392	1.480 (0.382, 5.735)	0.571
Complement C4	1.411	4.102 (0.554, 30.362)	0.167
Creatinine	-0.006	0.994 (0.979, 1.009)	0.402
HDL	-0.020	0.980 (0.516, 1.862)	0.951
VZV-IgA	-0.220	0.802 (0.631, 1.020)	0.072
VZV-IgG	-0.006	0.994 (0.931, 1.061)	0.855
VZV-IgM	0.041	1.042 (0.685, 1.585)	0.849
LDL	-0.154	0.857 (0.651, 1.129)	0.273

**Figure 2:**
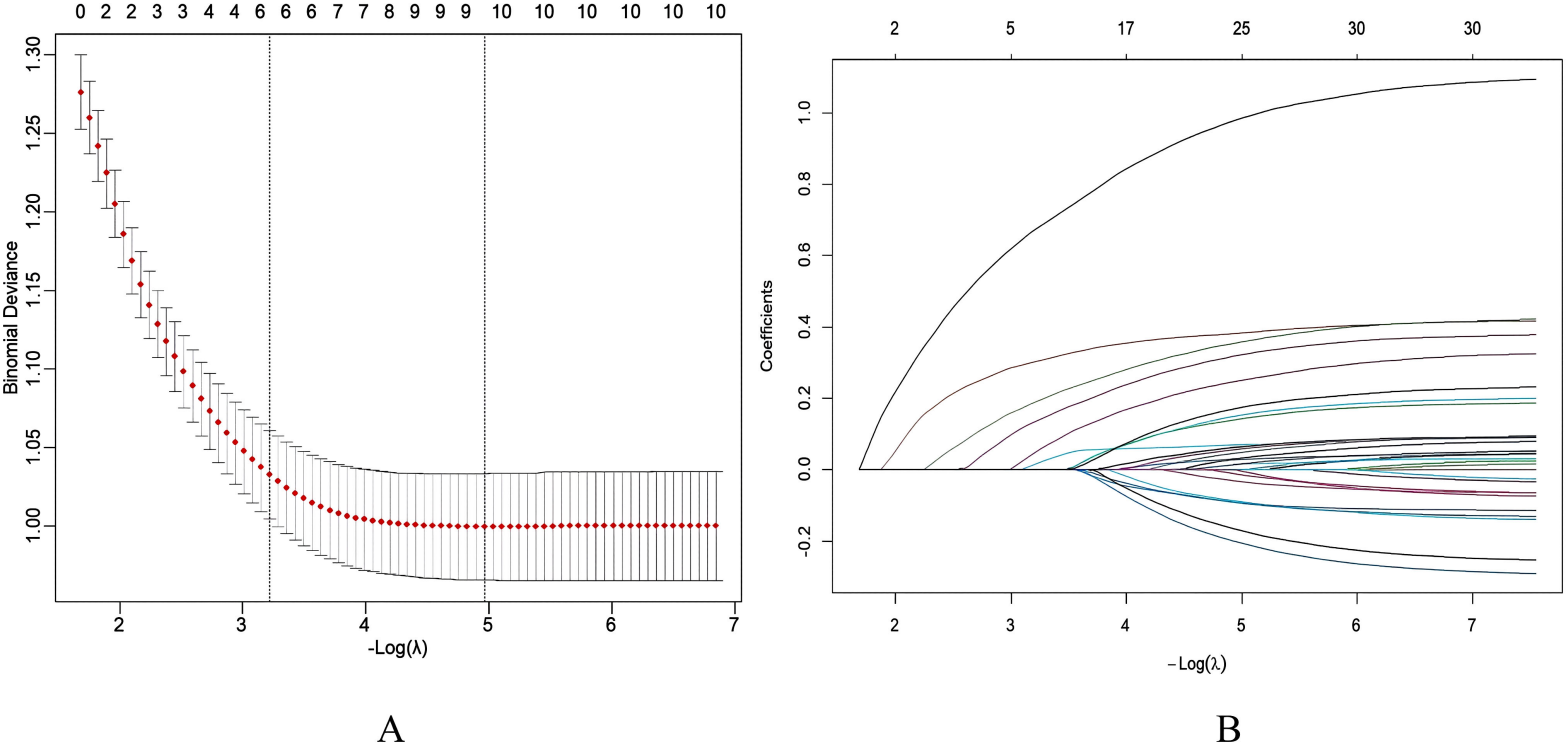
Selection plot of the optimal penalty factor λ **(A)** and elastic net regression coefficient path plot **(B)**.

LASSO regression showed that when the penalty factor λ took the optimal value of 0.0278 corresponding to the 1-se rule, the model incorporating 6 variables with non-zero coefficients were the optimal predictive variable combination (**Figure 2A**). The simultaneously plotted elastic net regression coefficient path map further verified that the optimal model generalizability was achieved (**Figure 2B**). Based on the above analysis, 6 core predictive variables were finally screened out, namely age, NRS score, time to treatment, SIRI, location_X2 (thoracodorsal region), and NAR, as shown in **Table 5**, **6**.

**Table 5:** Core predictive factors and coefficients screened by LASSO regression.

Item	Regression coefficient	Penalty factor λ
(Intercept)	-0.835	0.0278
Age	0.752	0.0278
NRS score	0.331	0.0278
Time to treatment	0.236	0.0278
SIRI	0.187	0.0278
location_X2 (thoracodorsal region)	0.115	0.0278
NAR	0.0536	0.0278

**Table 6:** Optimal cutoff values of core predictive variables.

Term	Optimal cutoff value
Age	63 years old
NRS score	4 points
Time to treatment	10 days
SIRI	1.04
NAR	1.74

**Figure 3:**
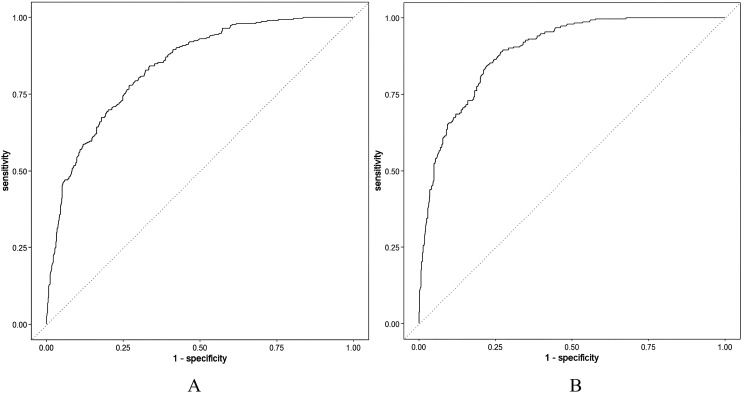
ROC curve of 5-variable model without SIRI **(A)** and 6-variable model incorporating SIRI **(B)**. ROC, receiver operating characteristic; The gray dashed diagonal line represents the reference line of a random guess model with an AUC of 0.5.

Receiver operating characteristic (ROC) curve analysis showed that the AUC of the 6-variable predictive model incorporating SIRI was 0.890 (95% CI 0.869–0.911) in the training set, which was significantly higher than that of the 5-variable model without SIRI (AUC = 0.838, 95% CI 0.811–0.865) (**Figures 3A, B**).

### Construction and internal validation of machine learning models

**Figure 4:**
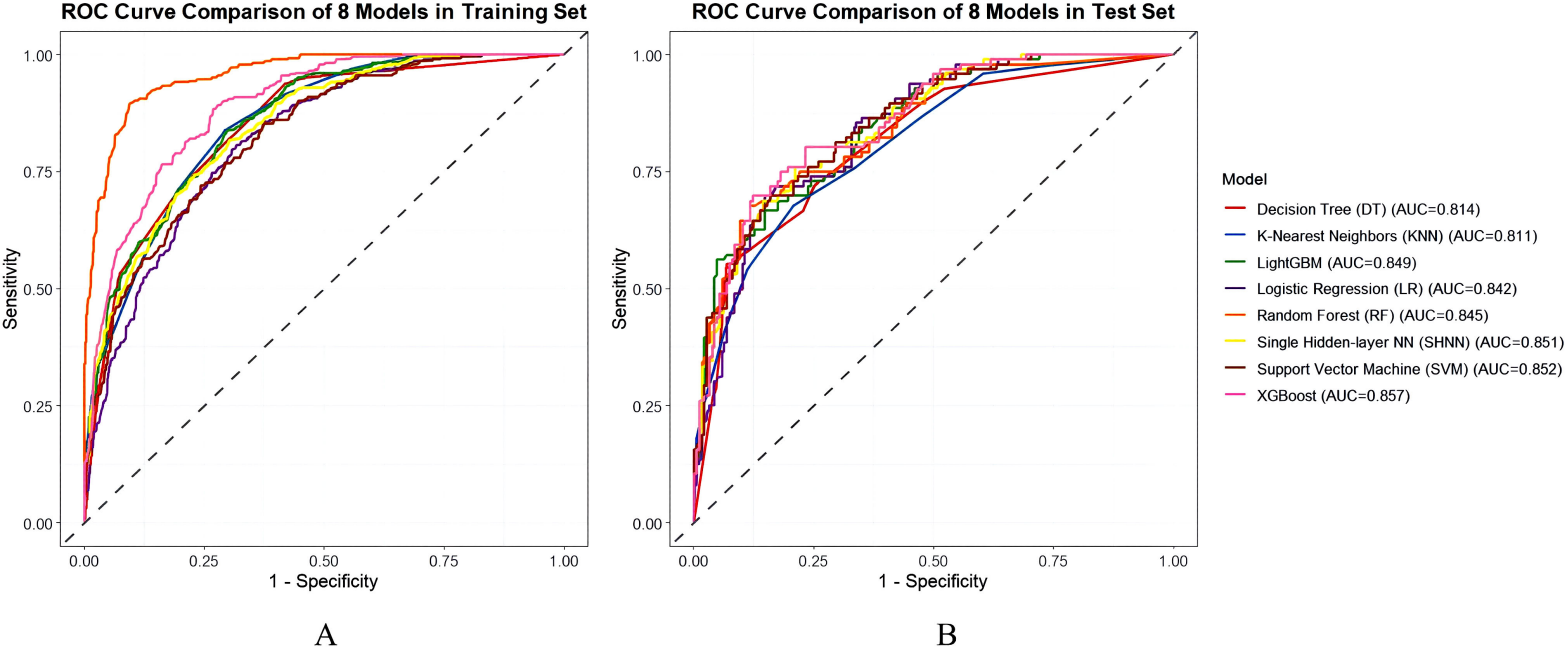
Comparison of ROC curves of 8 models for predicting PHN risk in training set **(A)** and in test set **(B)**.

To comprehensively evaluate model performance, we reported 11 metrics including AUC, sensitivity, specificity, accuracy, PR-AUC, Brier score, F1 score, NPV, PPV, Youden index, and Kappa coefficient. The performance indicators of 8 models in the training set and test set are shown in **Table 7**, and the comparison of ROC curves is shown in **Figures 4A, B**. The results showed that the XGBoost, LightGBM, and RF models had the best predictive performance in the training set. Among them, the difference in AUC between the training set and the test set of the RF model was 0.112 (>0.05), and the XGBoost model was only 0.032 (<0.05), with no obvious overfitting and excellent generalizability. In the test set, the XGBoost model had the highest AUC (0.857), accuracy (0.758), PR-AUC (0.776), and F1 score (0.693) among all models, with the lowest Brier score (0.139), while maintaining a good balance between sensitivity (0.812) and specificity (0.730).

**Table 7:** Comparison of performance indicators of 8 models in the training set and test set.

Model	Dataset	AUC	Sensitivity	Specificity	Accuracy	PR-AUC	Brier Score	F1 Score	Negative predictive value	Positive predictive value	Youden index	Kappa coefficient
XGBoost	Training set	0.889	0.842	0.781	0.801	0.791	0.133	0.740	0.907	0.659	0.623	0.583
	Test set	0.857	0.812	0.730	0.758	0.776	0.139	0.693	0.885	0.605	0.543	0.500
LR	Training set	0.819	0.807	0.683	0.725	0.684	0.162	0.663	0.875	0.562	0.490	0.442
	Test set	0.842	0.844	0.661	0.723	0.719	0.153	0.672	0.893	0.559	0.505	0.449
RF	Training set	0.957	0.898	0.731	0.787	0.821	0.092	0.739	0.934	0.627	0.629	0.568
	Test set	0.845	0.833	0.640	0.705	0.750	0.147	0.656	0.883	0.541	0.474	0.418
SVM	Training set	0.826	0.896	0.593	0.695	0.760	0.165	0.664	0.918	0.528	0.488	0.417
	Test set	0.852	0.849	0.628	0.702	0.715	0.161	0.657	0.892	0.535	0.477	0.417
SLNN	Training set	0.847	0.818	0.658	0.712	0.684	0.172	0.655	0.877	0.547	0.476	0.424
	Test set	0.851	0.823	0.630	0.695	0.716	0.182	0.645	0.875	0.530	0.453	0.398
KNN	Training set	0.853	0.839	0.706	0.751	0.753	0.182	0.693	0.897	0.590	0.545	0.493
	Test set	0.811	0.760	0.661	0.695	0.716	0.213	0.627	0.845	0.533	0.422	0.382
DT	Training set	0.848	0.937	0.581	0.700	0.747	0.142	0.677	0.948	0.530	0.517	0.435
	Test set	0.814	0.906	0.513	0.646	0.711	0.151	0.633	0.915	0.486	0.419	0.346
LightGBM	Training set	0.858	0.832	0.733	0.766	0.774	0.139	0.704	0.896	0.733	0.564	0.518
	Test set	0.849	0.833	0.661	0.719	0.739	0.153	0.667	0.887	0.556	0.495	0.441

PR-AUC, Precision-Recall Area Under the Curve; PPV, Positive Predictive Value; NPV, Negative Predictive Value; Sensitivity (Recall) = TP/(TP + FN); Specificity = TN/(TN + FP); Accuracy = (TP + TN)/(TP + TN + FP + FN); F1 Score = 2 × (Sensitivity × Positive Predictive Value)/(Sensitivity + Positive Predictive Value); Negative Predictive Value (NPV) = TN/(TN + FN); Positive Predictive Value (PPV, Precision) = TP/(TP + FP; Youden Index = Sensitivity + Specificity - 1.

**Figure 5:**
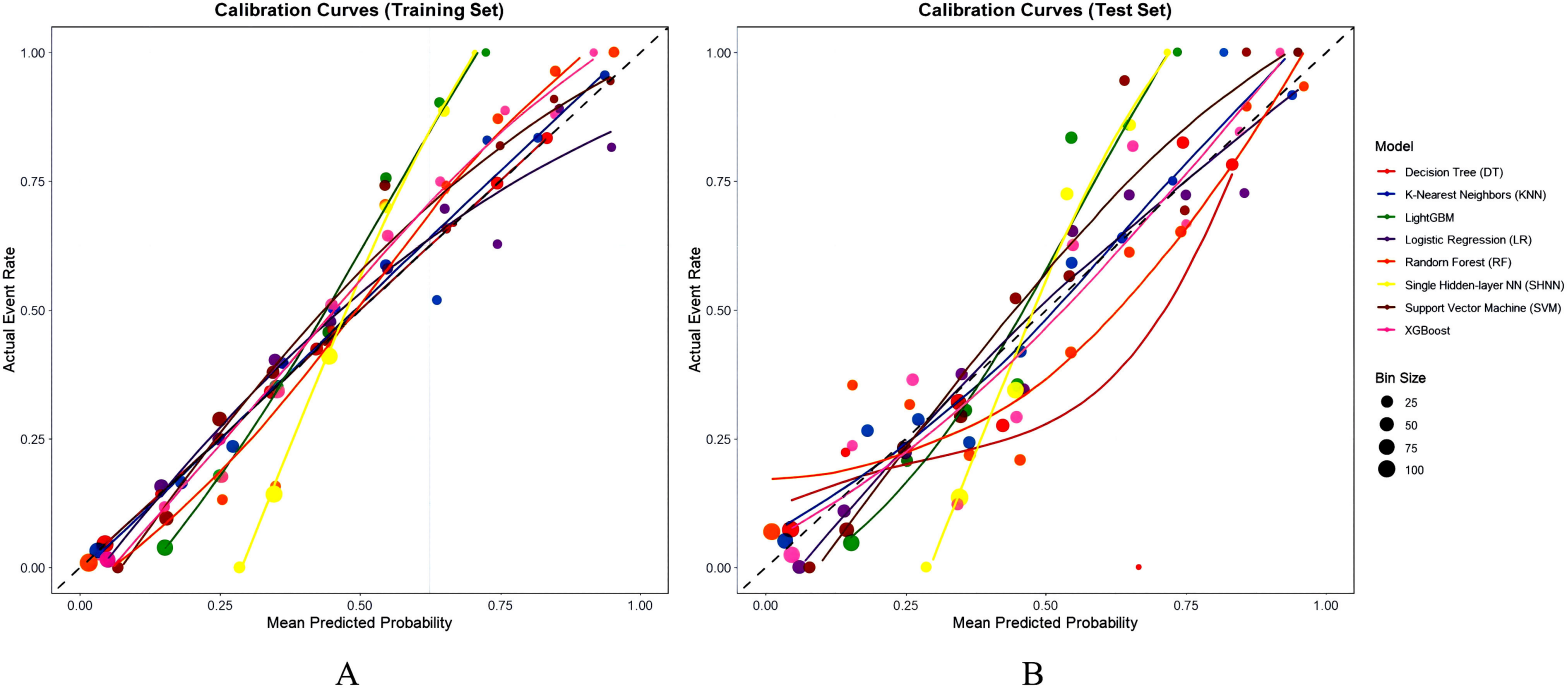
Calibration curves of 8 models for predicting PHN risk in training set **(A)** and in test set **(B)**. The black dashed diagonal line is the 45° perfect calibration line, which represents complete consistency between the predicted probability and the actual incidence rate. The scatter points are the calibration points of each probability bin, and the size of the scatter points corresponds to the sample size in the bin. The legends on the right of the plots indicate the names of the prediction models and the mapping between point size and bin sample size.

**Figure 6:**
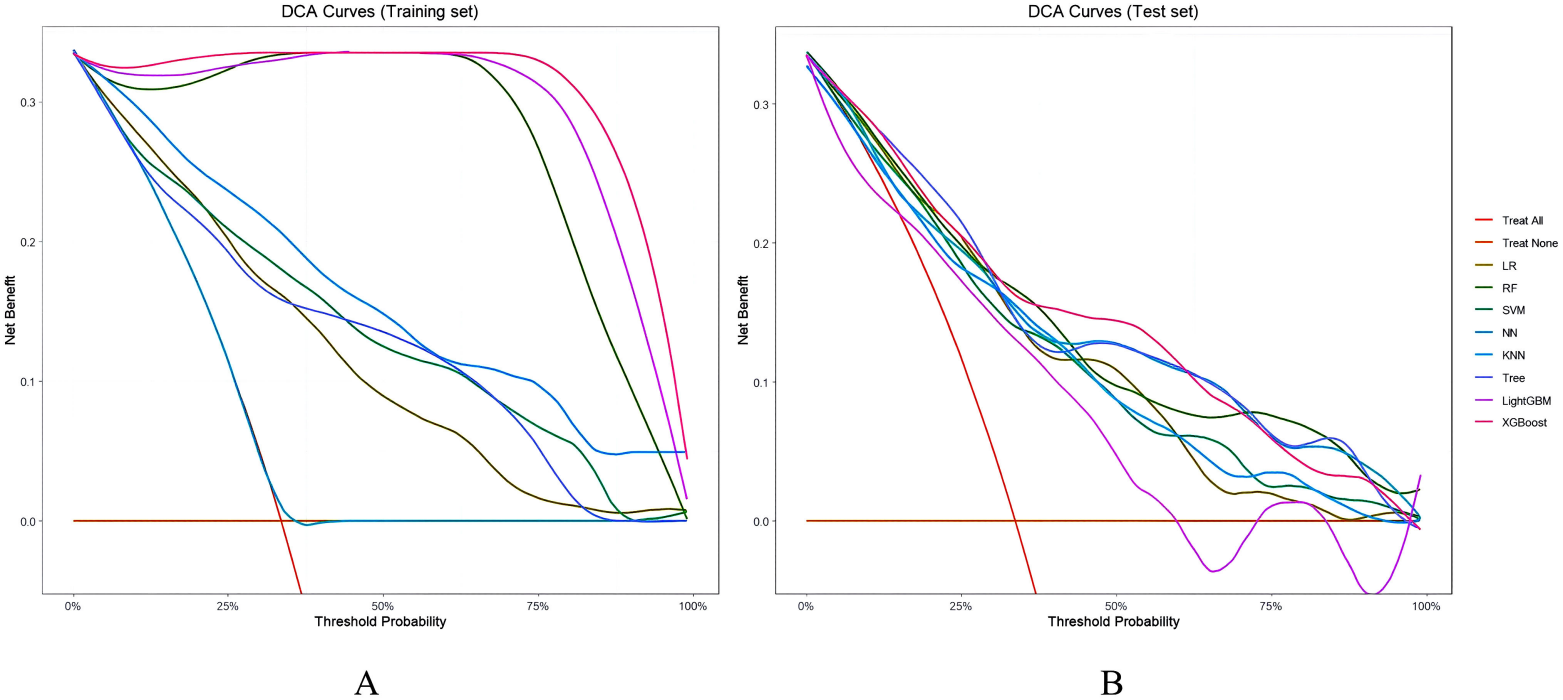
DCA plot of 8 models for predicting PHN risk in training set **(A)** and in test set **(B)**. The “Treat All” curve represents the extreme strategy of implementing preventive intervention for all patients, while the “Treat None” curve represents the extreme strategy of implementing no preventive intervention for any patient.

The calibration curves of the 8 models in the train set and test set are shown in **Figures 5A, B**. Among them, the calibration curve of the XGBoost, LR, and KNN models had the best fit with the 45° ideal calibration line. The results of DCA are presented for both the training set and test set is shown in **Figures 6A, B**. The XGBoost, RF and LightGBM models showed the optimal net benefit in the training set. In the test set, the XGBoost curve consistently remained above the two extreme strategy curves (“Treat All” and “Treat None”) without negative net benefit throughout the threshold range, indicating the widest clinical applicability.

Despite slight differences among the models, comprehensive consideration showed that the order of importance was age, time to treatment, SIRI, NRS score, location_X2 (thoracodorsal region), and NAR, and the predictive effect of each variable on PHN was positively correlated, as shown in **Supplementary Table 3**. After systematic and comprehensive evaluation, XGBoost, RF, and LightGBM were screened as the top 3 models with the best overall comprehensive performance.

### Independent external validation of the optimal model

**Figure 7:**
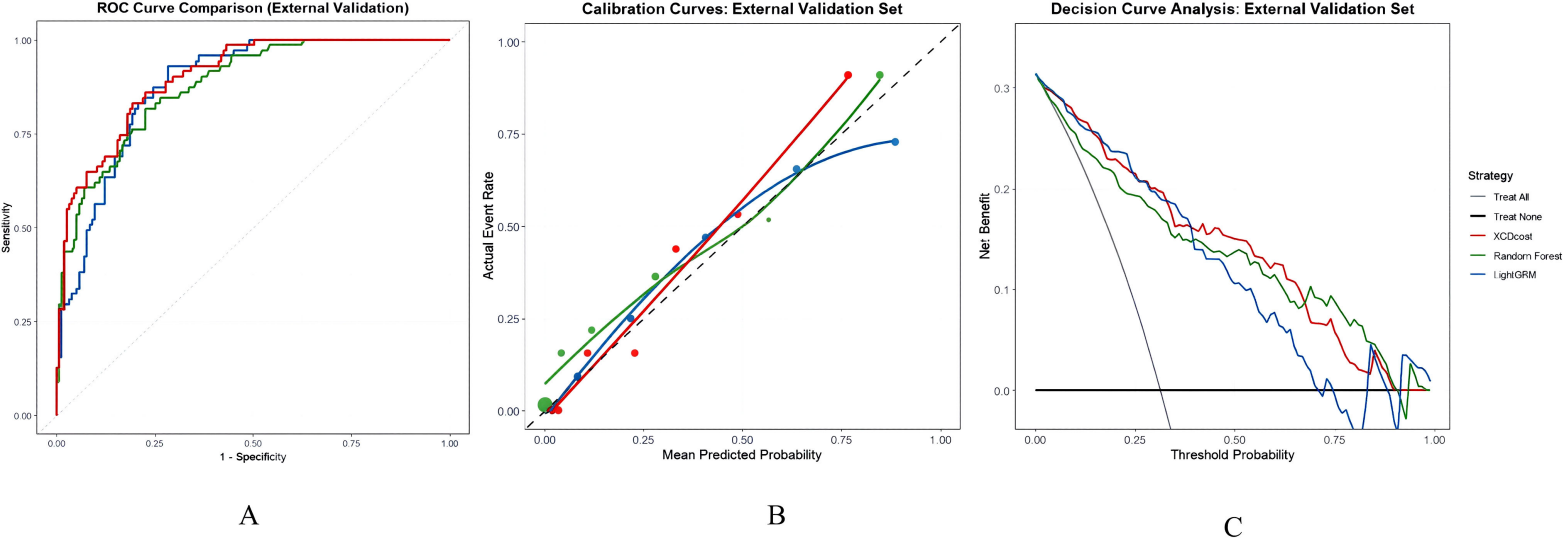
ROC curves **(A)**, calibration curves **(B)**, and DCA **(C)** comparison of the XGBoost, LightGBM, and RF models in the independent external validation cohort.

The performance indicators of 3 models are shown in **Table 8**. The discrimination ability of the three models was evaluated by ROC curves, with the XGBoost model achieving the highest area under the AUC of 0.900, followed by LightGBM (AUC = 0.879) and RF (AUC = 0.877) (**Figure 7A**). Calibration curves demonstrated that the XGBoost model had the optimal consistency between predicted probabilities and actual observed incidence of PHN, with its curve closest to the ideal diagonal reference line (**Figure 7B**). DCA further revealed that the XGBoost model provided the highest clinical net benefit across the majority of clinically relevant threshold probability ranges, confirming its superior clinical application value (**Figure 7C**).

**Table 8:** Comparison of performance indicators of the 3 optimal models in the external validation set.

Model	AUC	Sensitivity	Specificity	Accuracy	PR-AUC	Brier Score	F1 Score	Negative predictive value	Positive predictive value	Youden index	Kappa coefficient
XGBoost	0.900	0.746	0.877	0.836	0.851	0.119	0.741	0.883	0.736	0.624	0.622
RF	0.877	0.803	0.768	0.779	0.789	0.123	0.695	0.895	0.613	0.571	0.526
LightGBM	0.879	0.690	0.852	0.801	0.804	0.119	0.685	0.857	0.681	0.542	0.540

The results showed that the XGBoost model still maintained the optimal and robust predictive performance in the external validation set, with an AUC of 0.900. Meanwhile, its accuracy, specificity, F1 score, Youden index, and Kappa coefficient were the highest among the 3 models, with the lower Brier score, further confirming the good cross-center generalizability of the model, and was thus identified as the final optimal model for PHN risk prediction.

### Incremental predictive value of SIRI

To further demonstrate the incremental predictive value of SIRI, we constructed and compared four models:(1) Clinical-only model: Age, NRS score, time to treatment, rash location (thoracodorsal region); (2) Laboratory-only model: SIRI, NAR; (3) Clinical + SIRI model: Clinical-only model plus SIRI; and (4) Final XGBoost model: All 6 core variables (age, NRS score, time to treatment, rash location, SIRI, NAR). As shown in **Table 9**, adding SIRI to the clinical-only model resulted in substantial and statistically significant improvements in both the training set (AUC from 0.850 to 0.881) and internal test set (AUC from 0.814 to 0.856). Notably, the clinical + SIRI model exhibited markedly improved robustness across all three cohorts: the difference in AUC between the internal test set and external validation set was reduced from 0.090 for the clinical-only model to only 0.045, indicating that SIRI effectively mitigated overfitting and enhanced the generalizability of the model. In the independent external validation set, the clinical + SIRI model maintained excellent discriminative ability with an AUC of 0.901, nearly identical to the clinical-only model (0.904), while using only one additional routinely available laboratory parameter. The final XGBoost model incorporating both SIRI and NAR achieved the most balanced performance across all cohorts, with consistent high discriminative ability and minimal performance degradation between internal and external validation, demonstrating that combining traditional clinical characteristics with inflammatory biomarkers produces a more robust and clinically generalizable prediction tool.

**Table 9:** Performance comparison of four models.

Model	Training set AUC (95% CI)	Internal test set AUC (95% CI)	External validation set AUC (95% CI)
Clinical-only model	0.850 (0.824–0.877)	0.814 (0.765–0.863)	0.904 (0.865–0.944)
Laboratory-only model	0.669 (0.630–0.707)	0.698 (0.633–0.764)	0.717 (0.646–0.788)
Clinical + SIRI model	0.881 (0.858–0.903)	0.856 (0.812–0.900)	0.901 (0.862–0.940)
Final XGBoost model	0.889 (0.868–0.910)	0.857 (0.813–0.901)	0.900 (0.860–0.940)

### Interpretability analysis of the optimal model

**Figure 8:**
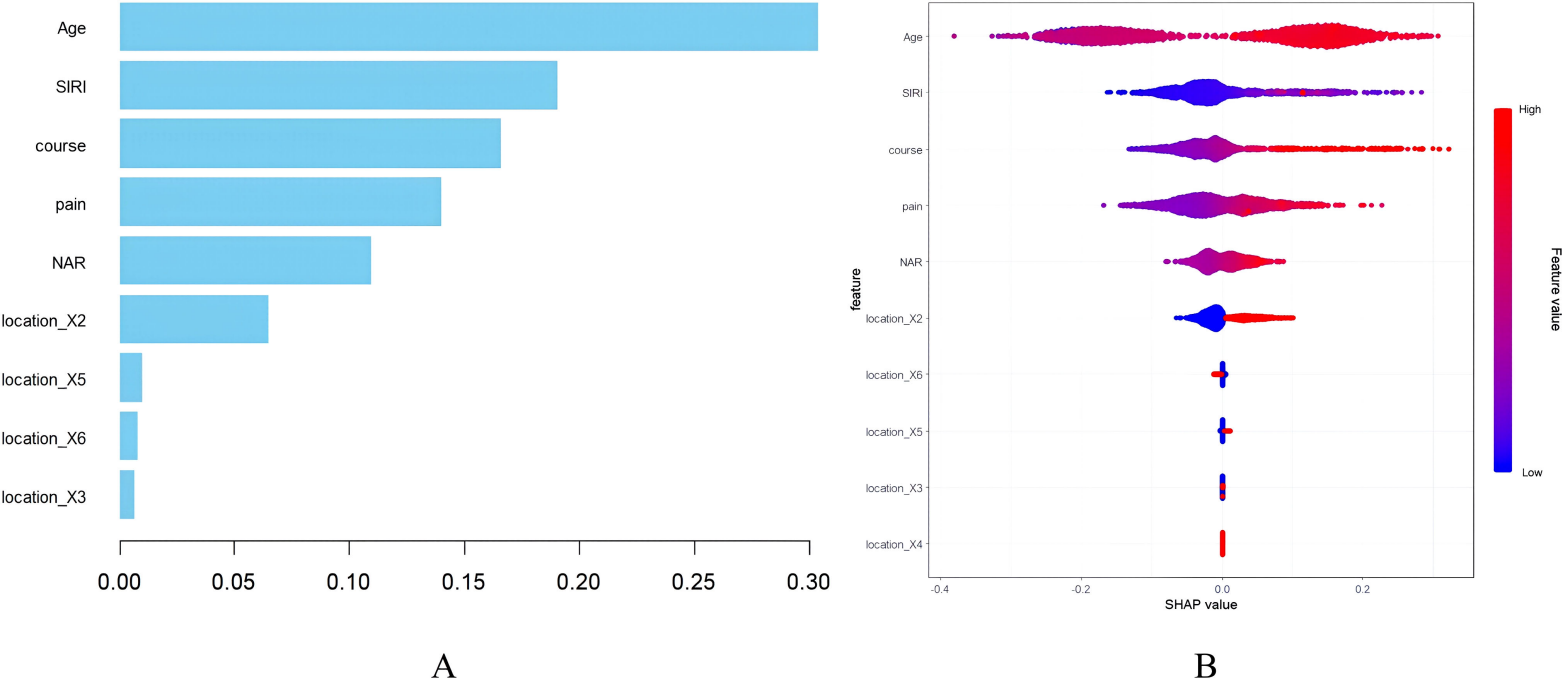
Feature importance analysis plot **(A)** and SHAP global beeswarm plot **(B)** of the XGBoost model for predicting PHN risk. pain = Numerical Rating Scale score; course = time to treatment.

The feature importance bar plot showed that age, SIRI, time to treatment, NRS score, NAR, and rash location (thoracodorsal region) were the top 6 features with the highest contribution to the model prediction results, among which SIRI ranked second in contribution, accounting for approximately 20.1%, as shown in **Figure 8A**. The SHAP global beeswarm plot intuitively presented the high values of each feature corresponded to positive SHAP values, indicating that they were associated with an increased risk of PHN occurrence, as shown in **Figure 8B**.

### Construction and validation of risk scoring table

The final established scoring system had a theoretical total score range of 5 to 24 points, with an actual observed total score range of 6 to 24 points in the study population. The optimal cut-off value of the total risk score was determined as 18 points according to the maximum Youden index of the ROC curve in the training set, as shown in **Table 10**; based on this cut-off value and the distribution characteristics of the total score, we further stratified the study population into three risk strata, as shown in **Table 11**.

**Table 10:** Simplified clinical risk scoring table for PHN prediction.

Indicator	Grading criteria	Score
Age (years)	[18,50)	1
	[50,60)	3
	[60,70)	5
	[70,80)	6
	≥80	8
NRS score (points)	0	0
	(1, 3)	1
	[4,6]	2
	[7,10]	3
Time to treatment (days)	[0,4)	1
	[4,6)	2
	[6,10)	3
	≥10	4
SIRI	[0.14,0.6)	1
	[0.6,0.95)	2
	[0.95,1.59)	3
	≥1.59	4
NAR	[0.72,1.57)	1
	[1.57,1.89)	2
	≥1.89	3
Rash location	Cephalic-facial, cervicoscapular, Lumbabdominal, upper limb, lower limb	1
	Thoracodorsal	2

**Table 11:** Risk stratification results by risk score.

Risk stratum	Number of samples (n)	Number of PHN cases (n)	PHN incidence rate (95% CI)	Total score range (points)	Mean total score (points)
Low risk	234	13	5.6% (2.6-8.5%)	6–12	11
Moderate risk	463	163	35.2% (30.9-39.6%)	13–17	15
High risk	153	109	71.2% (64.1-78.4%)	18–24	19

**Figure 9:**
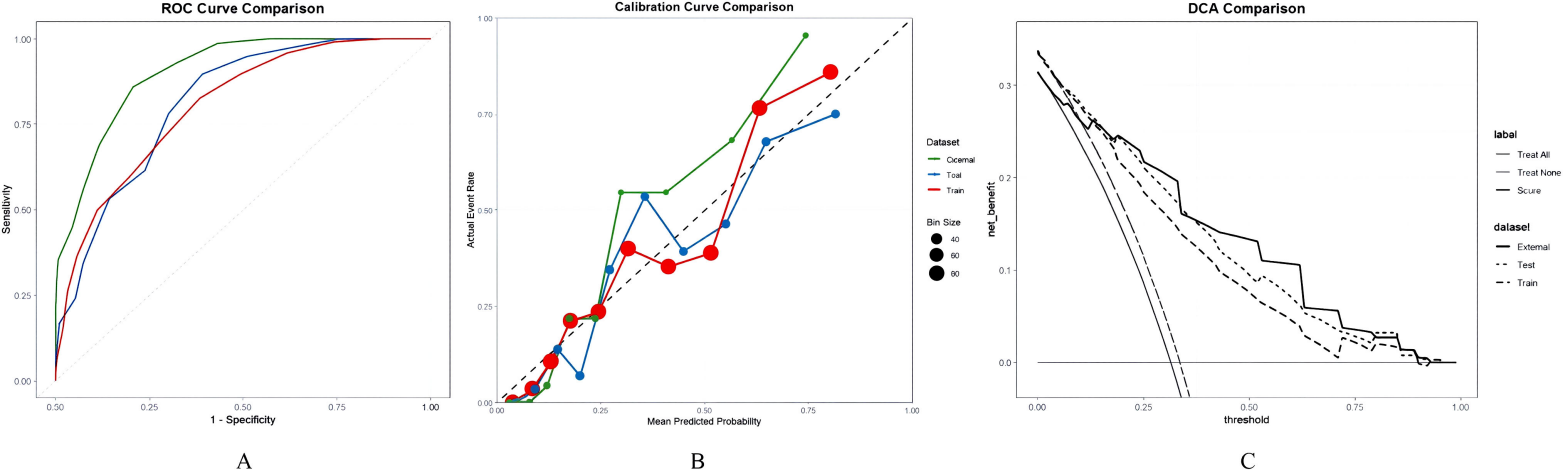
ROC curves **(A)**, calibration curves **(B)**, and DCA **(C)** comparison of the PHN risk scoring system in three cohorts.

The developed clinical risk score for PHN exhibited robust and favorable predictive performance, with an AUC of 0.803 in the training cohort, 0.813 in the internal test cohort, and 0.904 in the external validation cohort (**Figure 9A**), indicating excellent discriminative power for PHN risk stratification and cross-center generalizability. Calibration curves demonstrated favorable consistency between the predicted PHN risk and the actual observed incidence across all three cohorts. The curve for the independent external validation cohort showed good alignment with the ideal diagonal line in the clinically most relevant low-to-moderate predicted probability range (0 to 0.65). A slight overestimation of the actual incidence was observed at the high predicted probability range (>0.65), indicating good generalizability (**Figure 9B**). Decision curve analysis also demonstrated that the risk score provided a significantly higher clinical net benefit than the default “treat all patients” and “treat none” strategies across the threshold probability range of 0.05 to 0.9 in all three cohorts, and maintained a superior net benefit in the independent external validation cohort, confirming that the score has favorable clinical utility to guide personalized preventive intervention for PHN in clinical practice (**Figure 9C**).

## Discussion

The incidence of PHN in the population included in this study was 37.55%, which was slightly higher than the 29.8% previously reported in domestic epidemiological studies. The main reason is that the two centers included in this study are all provincial tertiary grade A general hospitals, and the enrolled patients had more severe acute phase pain and a higher proportion of elderly patients, who are high-risk groups for PHN. Therefore, the incidence rate is slightly higher than the epidemiological data of the community population.

The latency and reactivation of VZV are highly dependent on the cell-mediated immune function of the body, and the neuroinflammation and immune injury caused by VZV are the pathological basis for the development and progression of PHN. The 6 indicators finally included in this study fully cover the pathological links of PHN occurrence. Among them, age is a well-recognized core risk factor for PHN, and the results of this study are consistent with previous studies. Research (16) indicates that the risk of PHN onset (defined as pain ≥90 days post-rash onset) increases significantly with age, with individuals over 50 having 8–10 times the risk of younger populations, and those aged 65 and above experiencing approximately 5 times the incidence rate of younger cohorts (17). Its internal mechanism is closely related to age-related T cell function decline, reduced VZV clearance efficiency, and continuous aggravation of neuroinflammatory injury (18). The degree of acute phase pain is a direct clinical manifestation of nerve injury caused by viral replication. This study confirmed that the higher the NRS score, the significantly higher the risk of PHN, which is consistent with the results of many previous prospective cohort studies, further verifying the direct correlation between the degree of acute neuroinflammatory injury and chronic neuropathic pain. Time to treatment, as the only modifiable risk factor in this study, has been confirmed to be positively correlated with PHN risk by many studies (11, 19). Delayed consultation will miss the optimal window period for antiviral and pain intervention, leading to sustained viral replication and aggravation of the neuro-injury cascade. For rash location, some previous studies suggested that patients with cephalic-facial HZ have a higher risk of PHN, while the results of this study showed that thoracodorsal HZ is an independent risk factor for PHN. This difference may be due to the fact that thoracodorsal HZ mostly involves more extensive dermatomes and a wider range of nerve injury, but patients have higher pain tolerance and a higher proportion of delayed consultation, which ultimately leads to an increased risk of PHN. Its specific mechanism still needs to be further clarified by large-sample studies in the future.

The core innovation of this study is that it is the first to systematically incorporate the novel composite inflammatory markers SIRI and NAR into the PHN risk prediction system, filling the gap in previous PHN prediction studies that lack objective biomarkers integrating inflammation, immunity, and nutritional status. Previous studies have confirmed that SIRI has excellent value in the prognostic prediction of chronic postoperative pain (20), various malignant tumors (9), cardiovascular diseases (8, 21), and infectious diseases (7, 22). In this study, multivariate binary LR confirmed for the first time that SIRI is an independent risk factor for PHN (OR = 1.448, 95% CI 1.119–1.874, *P* = 0.005), with an 44.8% increased risk of PHN per unit increase in SIRI, and its independent predictive value remained stable after adjusting for confounding factors such as acute phase intervention measures. Meanwhile, SHAP showed that SIRI ranked second in contribution to model prediction, further verifying its core value in PHN risk assessment. In addition, SIRI can be calculated only through routine blood tests, which is convenient for detection. We recommend that SIRI detection be included in the routine assessment of all patients with HZ to provide a reference for the early prediction of PHN. NAR, as a composite marker integrating inflammatory activation and nutritional antioxidant capacity, its neutrophil percentage directly reflects the degree of systemic inflammatory activation, and serum albumin reflects the body’s nutritional status and antioxidant damage capacity. The combination of the two can assess the potential risk of nerve injury from both inflammation and nutrition aspects. Previous studies have confirmed that NAR has good application value in the prognostic assessment of infectious (23, 24) and inflammatory disorders (25). In this study, the serum NAR level of PHN patients was slightly higher than that of non-PHN patients. The internal mechanism is that the systemic inflammatory response caused by VZV infection can activate and proliferate neutrophils, while the persistent inflammatory state will increase the body’s nutritional consumption and weaken the neuroprotective ability of albumin. Together, they aggravate nerve injury and pain sensitization, and ultimately increase the risk of PHN. Although both SIRI and NAR contain neutrophil-related information, they reflect distinct pathophysiological aspects of PHN development with no biological redundancy. SIRI simultaneously integrates two innate immune effector cells (neutrophils and monocytes) and the adaptive immune component (lymphocytes), comprehensively reflecting the overall inflammatory-immune homeostasis imbalance caused by VZV infection. In contrast, NAR integrates inflammatory activation and nutritional antioxidant capacity. The combination of the two can assess the potential risk of nerve injury from both the immune-inflammatory response and nutritional reserve aspects, providing complementary predictive information. It is worth noting that in this study, univariate analysis showed that previously reported risk factors such as immunosuppressive status and diabetes history were significantly correlated with PHN occurrence, but their coefficients were compressed to 0 after dimensionality reduction by LASSO regression. It is speculated that the reason is that the effect of these factors on PHN can be largely reflected by direct indicators such as SIRI and NAR. Meanwhile, affected by collinearity and sample population characteristics, their independent predictive efficiency is weakened. The true association between them and PHN still needs further verification by more high-quality studies.

Compared with the traditional LR model widely used in previous PHN prediction studies, the XGBoost machine learning algorithm used in this study shows significant performance advantages. Although LR has good interpretability, it can only capture the linear relationship between variables, and is difficult to handle complex nonlinear associations and variable interactions in clinical data, which is the core reason why the AUC of most previous PHN prediction models is mostly concentrated in 0.70–0.80 with limited predictive performance. As a gradient boosting ensemble algorithm, XGBoost can automatically mine the complex interaction effects between variables through iterative fitting of residuals, such as the synergistic amplification effect of age and inflammatory indicators on PHN risk in this study, and ultimately achieve better prediction accuracy. Aiming at the problem of limited clinical promotion caused by the “black box effect” of machine learning models, this study conducted a comprehensive interpretability analysis of the model through SHAP values, clarified the contribution and influence direction of each variable on the prediction results, helped clinicians clearly understand the decision-making basis of the model, and greatly improved the clinical acceptance of the model. More importantly for clinical practice, based on SHAP values and the optimal cutoff values of variables, this study developed a simplified risk scoring table with only 6 indicators. This scoring table does not require complex calculation tools, and can complete rapid bedside risk stratification only through medical history inquiry and routine blood and biochemical tests on admission, solving the pain point that most previous machine learning models are difficult to promote in primary medical institutions. The results of this study showed that the scoring table maintained stable predictive performance in the training set, internal test set, and external validation set, and the model had a high negative predictive value of 94.4%, which can accurately exclude low-risk patients and effectively reduce unnecessary overtreatment. Meanwhile, with 18 points as the optimal cutoff value, it can effectively identify high-risk patients with PHN. For high-risk patients with a score ≥18 points, early intensive pain management, nerve block and other preventive interventions can be initiated clinically as soon as possible to minimize the risk of PHN. For low-risk patients, routine follow-up and symptomatic treatment can be taken to avoid potential risks caused by invasive procedures.

To further clarify the application limitations of this model, we conducted a characteristic analysis of patients with misclassification. A total of 1361 patients from two hospitals were finally included in this study, among which 285 patients had prediction deviations: 210 patients were judged as high risk by the model but did not actually develop PHN (false positives), and 75 patients were judged as low risk but actually developed PHN (false negatives). Most of the false positive patients had clinical risk factors similar to those of the high-risk population, but had milder acute phase pain and lower SIRI levels, suggesting that in clinical practice, even if patients have risk factors such as advanced age and long time to treatment, if the acute phase pain and systemic inflammatory indicator levels are low, it is still necessary to make a prudent judgment combined with the patient’s overall situation to avoid overtreatment. Most of the false negative patients are elderly people, suggesting that for elderly patients in clinical practice, even if the model score is in the low-risk range, closer follow-up and more active pain intervention are still needed to reduce the risk of missed diagnosis of PHN. The incidence of PHN in the population included in this study is about 37.55%, and there is a certain degree of class imbalance in outcome events, which is also one of the potential reasons for the relative concentration of false negative cases. However, after multi-step variable screening and model optimization, the final model still maintained a sensitivity of 84.2% and an overall classification accuracy of 80.1%, with an F1 score of 0.74. The overall performance is robust and can meet the needs of clinical PHN risk screening.

Although this study systematically verified the independent predictive value of SIRI and constructed a prediction model and clinical scoring tool with excellent performance based on a multi-center prospective cohort design, there are still some limitations. First, the population generalizability of this study has certain limitations. Although a multi-center study design was adopted and independent external validation was completed, all participating centers are from Zhejiang Province, and the extrapolation of the model to other regions in China and even different ethnic groups still needs further verification. Second, the follow-up endpoint of this study was set at 90 ± 7 days after HZ onset, which is in line with the international general diagnostic criteria for PHN, but the model’s predictive performance for long-term PHN 6 months, 1 year or more after onset cannot be evaluated. Meanwhile, only a single peripheral blood sample was collected at the time of patient admission, and the change trend of inflammatory indicators during the disease process was not dynamically tracked. Subsequent studies can collect multi-timepoint dynamic blood samples to explore the predictive value of dynamic changes in inflammatory indicators for PHN, and combine with other biomarkers such as nerve injury marker S100-β and metabolomic characteristics to further improve the prediction accuracy of the model. Third, this study did not include HZ vaccination status as a predictive factor, mainly due to the high missing rate of vaccination history records in the study population and insufficient data integrity. With the gradual popularization of HZ vaccination in China, subsequent studies can include vaccination history as a key variable into the model to further improve the risk assessment tool. Finally, during the follow-up period, this study observed that some patients received special pain intervention treatments such as nerve block, spinal nerve root radiofrequency ablation, and trigeminal radiofrequency lesioning. Given that the primary purpose of this study was to develop an early prediction model that can be used at the time of first visit (before any treatment decisions are made), special treatments were not included as predictive factors in the initial model development. However, these patients generally had higher NRS scores, with a mean of 5–6 points, and are themselves high-risk groups for PHN with higher willingness to seek medical treatment. This raises the question of whether the special intervention treatment received by these patients can offset their own risk of PHN and change the PHN outcome. To assess the potential confounding effect of special pain interventions, we conducted two additional analyses. We first performed a subgroup analysis of patients with model misclassification, and found that among 210 false positive patients who were predicted to develop PHN but did not actually experience the outcome, 10.95% received special pain intervention treatments during follow-up, while only 6.67% of 75 false negative patients who were predicted to be low-risk but eventually developed PHN received relevant interventions, suggesting that some high-risk patients may change the final PHN outcome through special treatment, this speculation still lacks direct evidence support. Furthermore, we conducted a sensitivity analysis by including “whether received special pain intervention treatment” as an additional covariate into the multivariate binary logistic regression model, and the results showed that after adjusting for age, NRS score, time to treatment, rash location, and receipt of special pain intervention, SIRI remained a statistically significant independent risk factor for PHN with an almost unchanged odds ratio (OR = 1.459, 95% CI 1.127–1.889, *P* = 0.004) compared with the original unadjusted analysis (OR = 1.448, 95% CI 1.119–1.874, *P* = 0.005), indicating that its predictive value was not significantly interfered by special treatment. Combined with the analysis results of this study, although special treatment may have a potential impact on the PHN outcome of some patients, it did not interfere with the independent predictive value of SIRI. Therefore, there is no need to exclude these patients at the initial stage of the study, which also preserves the integrity of the study sample and reduces selection bias. However, the specific effect and internal mechanism of special treatment on PHN outcome still need to be further clarified by subsequent large-sample, targeted clinical studies.

Despite the above limitations, this study still has significant methodological and clinical application advantages. First, this study was conducted based on a prospective cohort design. All enrolled patients completed clinical data collection and specimen testing through standardized procedures, with high data integrity. Compared with retrospective studies, it greatly reduces recall bias and selection bias, and the study results have higher credibility. Second, this study is the first to systematically confirm the independent predictive value of SIRI and NAR for PHN, making up for the deficiency of previous PHN prediction models that only rely on clinical symptoms and lack objective quantitative inflammatory markers. Moreover, both indicators can be obtained only through routine clinical testing, which is convenient and low-cost, with extremely high clinical accessibility. Third, through the systematic comparison of 8 machine learning algorithms, this study screened out the XGBoost model with the best performance, which not only showed excellent performance in the training set and internal test set, but also confirmed the good generalizability of the model through external validation of an independent center, with an AUC of 0.900. The performance indicators remained stable, fully confirming the clinical applicability of the model. Fourth, through SHAP interpretability analysis, this study solved the “black box effect” of the machine learning model, and developed a simplified risk scoring table that is easy to use clinically, realizing the transformation from algorithm model to clinical tool. Even in primary medical institutions, rapid bedside stratification of high-risk PHN patients can be achieved, providing evidence-based basis for early precise clinical intervention.

## Conclusion

Based on a multicenter prospective cohort, this study is the first to confirm that the SIRI is an independent predictive biomarker for the occurrence of PHN. The XGBoost machine learning model constructed by integrating 6 core variables including SIRI, age, NRS score, time to treatment, rash location, and NAR shows excellent discriminative ability, calibration, and clinical net benefit in the training set, internal test set, and independent external validation set, with significantly better predictive performance than the traditional LR model and other machine learning models. The simplified risk scoring table developed based on SHAP can complete rapid risk stratification only with routine clinical and laboratory indicators, with strong clinical practicability. This study provides an evidence-based tool for the early risk assessment of PHN. Early active intervention is recommended for high-risk patients with a score ≥18 points to reduce the risk of PHN, realizes the early and accurate risk assessment of PHN, and provides an important basis for the formulation of clinical individualized treatment.

## Data Availability

The original contributions presented in the study are included in the article/supplementary material. Further inquiries can be directed to the corresponding author.

## Glossary

AUC: Area under the receiver operating characteristic curve
CI: Confidence Interval
CLR: C-reactive protein-to-lymphocyte ratio
CKD: Chronic Kidney Disease
COPD: Chronic Obstructive Pulmonary Disease;DCA, Decision Curve Analysis
DT: Decision Tree
eGFR: estimated ;lomerular Filtration Rate
HIV: Human Immunodeficiency Virus
hs-CRP: high-sensitivity C-reactive Protein
HDL: High-Density Lipoprotein
HZ: Herpes Zoster
IASP: International Association for the Study of Pain
ICMJE: International Committee of Medical Journal Editors
KNN: K-Nearest Neighbor algorithm
LASSO: Least Absolute Shrinkage and Selection Operator
LightGBM: Light Gradient Boosting Machine
LDL: Low-Density Lipoprotein
LR: Logistic Regression
MELD: Model for End-Stage Liver Disease
MICE: Multiple Imputation by Chained Equations
NAR: Neutrophil-to-Albumin Ratio
NLR: Neutrophil-to-Lymphocyte Ratio
NSE: Neuron-Specific Enolase
NRS: Numerical Rating Scale
NYHA: New York Heart Association
OR: Odds Ratio
PHN: Postherpetic Neuralgia
PR-AUC: Precision-Recall Area Under the Curve
RF: Random Forest
ROC: Receiver Operating Characteristic
SHAP: SHapley Additive exPlanations
SIRI: Systemic Inflammatory Response Index
SLNN: Single Hidden Layer Neural Network
SVM: Support Vector Machine
TRIPOD: Transparent Reporting of a multivariable prediction model for Individual Prognosis Or Diagnosis
VIF: Variance Inflation Factor
VZV: Varicella-Zoster Virus
VZV-IgA: Varicella-Zoster Virus-Immunoglobulin A
VZV-IgG: Varicella-Zoster Virus-Immunoglobulin G
VZV-IgM: Varicella-Zoster Virus-Immunoglobulin M
WBC: White Blood Cell
XGBoost: eXtreme Gradient Boosting
