# Supplementary material for: A multicenter prospective cohort study developing and validating a SIRI-based machine learning model and simplified risk score for predicting postherpetic neuralgia
Source: Front Immunol. 2026 Jun 5;17:1868650. doi: 10.3389/fimmu.2026.1868650 (PMC13279421; doi:10.3389/fimmu.2026.1868650)
Supplement: Supplementary file 1 [file Table1.docx]

**Supplementary Table 1.** Hyperparameter search ranges and adapted packages of 8 models

| Model | Core hyperparameters (after final optimization) | Code package |
| --- | --- | --- |
| XGBoost | mtry = 6, min_n = 6, tree_depth = 3, learn_rate = 0.00891, loss_reduction = 0.692, sample_size = 0.8 | xgboost |
| RF | mtry = 6, trees = 500, min_n = 50 | randomForest |
| KNN | neighbors = 11 | kknn |
| DT | tree_depth = 4, min_n = 8, cost_complexity = 0.000237 | rpart |
| SVM | cost = 32, rbf_sigma = 0.00316 | kernlab |
| LR | - | glm |
| LightGBM | tree_depth = 2, trees = 434, learn_rate = 0.0922, mtry = 6, min_n = 10, loss_reduction = 0.00103 | lightgbm |
| SLNN | hidden_units = 19.5, penalty = -1.5, epochs = 100, MaxNWts = 5000 | nnet |

Note: LR: logistic regression; DT: decision tree; RF: random forest; XGBoost: extreme gradient boosting; LightGBM: light gradient boosting machine; SVM: support vector machine; KNN: k-nearest neighbor; SLNN: single hidden-layer neural network; LR was fitted using the native generalized linear model (GLM) without hyperparameter tuning, as indicated by “-”.

**Supplementary Table 2.** Collinearity diagnosis results of candidate predictive factors

| Predictive Variable | VIF |
| --- | --- |
| Age | 1.579 |
| NRS score | 1.549 |
| Prodromal pain | 1.057 |
| Time to treatment | 1.592 |
| Hypertension | 1.238 |
| Diabetes | 1.108 |
| Sleep disorder | 1.224 |
| Immunosuppressive status | 1.104 |
| hs-CRP | 5.110 |
| WBC | 11.400 |
| Neutrophil percentage | 80.425 |
| Eosinophil percentage | 1.569 |
| Lymphocyte count | 7.932 |
| Monocyte count | 3.848 |
| Neutrophil count | 14.954 |
| NLR | 9.856 |
| CLR | 5.482 |
| NAR | 84.367 |
| SIRI | 7.940 |
| NSE | 1.125 |
| Albumin | 20.001 |
| Creatinine | 1.149 |
| Globulin | 3.352 |
| VZV-IgG | 3.226 |
| LDL | 1.193 |
| Rash location = thoracodorsal | 1.498 |
| Rash location = lumbabdominal | 1.393 |
| Rash location = cervicoscapular | 1.137 |
| Rash location = upper limb | 1.185 |
| Rash location = lower limb | 1.243 |

**Supplementary Table 3.** Ranking of importance of core predictive factors by 8 models

| Model | Importance ranking | | | | | |
| --- | --- | --- | --- | --- | --- | --- |
|  | 1 | 2 | 3 | 4 | 5 | 6 |
| SLNN | Age | Time to treatment | NRS score | Location_X2 | NAR | SIRI |
| SVM | Age | Time to treatment | NRS score | Location_X2 | NAR | SIRI |
| RF | Age | SIRI | NRS score | Time to treatment | NAR | Location_X2 |
| XGBoost | Age | SIRI | Time to treatment | NRS score score | Location_X2 | NAR |
| LR | Age | Time to treatment | NRS score | SIRI | Location_X2 | NAR |
| KNN | Age | NRS score | Time to treatment | Location_X2 | SIRI | NAR |
| LightGBM | Age | NRS score | SIRI | Time to treatment | NAR | Location_X2 |
| DT | Age | Time to treatment | SIRI | NRS score | NAR | Location_X2 |
